# Acute exacerbation of interstitial lung disease after transthoracic biopsy

**DOI:** 10.36416/1806-3756/e20230426

**Published:** 2024-09-16

**Authors:** Felipe Marques da Costa, Milena Tenorio Cerezoli, Christina Shiang, Bruno Lima Moreira, Augusto Kreling Medeiros

**Affiliations:** 1. Serviço de Pneumologia, Hospital Beneficência Portuguesa de São Paulo, São Paulo (SP) Brasil.; 2. Laboratório de Patologia Bacchi, São Paulo (SP) Brasil.; 3. BP Medicina Diagnóstica, Hospital Beneficência Portuguesa de São Paulo, São Paulo (SP) Brasil.

## TO THE EDITOR,

We report the case of an 80-year-old male with a recent diagnosis of advanced-stage lung adenocarcinoma, confirmed through transthoracic biopsy (TTB) in the right upper lobe, metastatic to bones and subcutaneous tissue. The patient had a 10-year history of rheumatoid arthritis, treated with adalimumab. He also had fibrotic rheumatoid arthritis-associated interstitial lung disease (RA-ILD) with combined pulmonary fibrosis and emphysema (CPFE) and fibrosis with a typical usual interstitial pneumonia (UIP) pattern; coronary artery disease requiring stent placement; symptomatic bradycardia requiring a pacemaker; and chronic kidney disease of undefined cause. He had a 20-pack-year smoking history and had ceased smoking 10 years prior. The patient was admitted to undergo a repeat TTB to collect material for a mutation panel and programmed death ligand 1 testing, given that the initial biopsy yielded insufficient material. Before the procedure, the patient had not undergone any oncological treatments. One week before this hospitalization, the patient experienced a small-volume hemoptysis episode, leading to a short (48-h) admission for bronchoscopy investigation. Signs of active bleeding from the right upper lobe were observed. Epinephrine was administered locally, and bronchial artery arteriography with selective embolization was performed, resulting in no further episodes of hemoptysis. No BAL or TTB was performed at that time.

**Figure 1:**
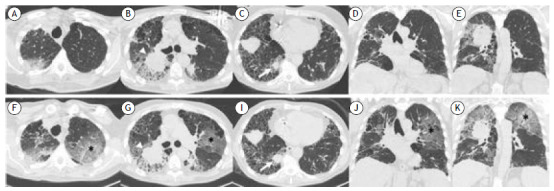
An initial chest CT (A-C: axial plane; D,E: coronal plane) showing a cavitary mass in the right lung (arrowhead), measuring 62 mm in diameter on its longest axis, centered in the posterior segment of the upper lobe and extending to the middle lobe and superior segment of the lower lobe. Note the other nodular lesions scattered throughout both lungs, as well as the ground-glass opacities surrounding those and the mass, which may correspond to perilesional hemorrhage. The images also show extensive (centrilobular and paraseptal) pulmonary emphysema, together with interstitial abnormalities characterized by reticular opacities, traction bronchiectasis/bronchiolectasis, and honeycombing, predominantly in the periphery of the lower lung fields (a typical UIP pattern on CT). Prominent lymph nodes are present in the mediastinum and pulmonary hila, as is a lytic lesion in the right scapula (not shown in the images). (F-J) A follow-up chest CT (F-H: axial plane; I,J: coronal plane), performed 12 days after the biopsy, revealing new ground-glass opacities scattered throughout both lungs, more extensively in the left upper lobe (asterisk), accompanied by sparse small consolidative foci, which may represent an acute exacerbation of the underlying interstitial lung disease. In addition, at least one nodular lesion in the right lower lobe (arrow) had increased in size in relation to what was seen on the initial CT.

On physical examination before the new lung biopsy, the RR was 16 breaths/min, the HR was 70 bpm, the SpO_2_ was 94% on room air, and there were basal crackles. Laboratory tests at admission showed abnormal renal function abnormalities (consistent with the preexisting condition of the patient) and findings consistent with anemia of chronic disease. An initial HRCT revealed a neoplastic mass in the right lung, together with CPFE (Figure 1, A-E). Ground-glass opacities surrounding the mass and other nodular lesions corresponded to perilesional hemorrhage identified within the context of hemoptysis. The patient underwent TTB, during which there were no complications and mechanical ventilation was not required. He was discharged the following day. The TTB confirmed a pattern of interstitial fibrosis with the spatial heterogeneity typically found in UIP, along with other areas of tumor infiltration into the lung parenchyma.

Ten days after the procedure, the patient returned to the emergency department with complaints of increased exertional dyspnea, a drop in SpO_2_ to 86% on room air, without fever, cough, sputum production, or upper airway symptoms. The RR was 28 breaths/min, the HR was 98 bpm, and pulmonary auscultation revealed worsening crackles up to the middle third of the lung. A repeat chest HRCT showed new diffuse bilateral ground-glass opacities (Figure 1, F-J). Initial diagnostic hypotheses included an infectious process or an acute exacerbation of ILD (AE-ILD).

At this point, empirical antibiotic therapy with piperacillin-tazobactam and azithromycin was initiated, and infectious causes were investigated. The results of blood cultures, urine culture, PCR for cytomegalovirus, an extended molecular panel for viruses and bacteria, and urine antigen tests for *Legionella* sp. and *Streptococcus* sp. were all unremarkable, as were those of tests for procalcitonin, galactomannan, B-type natriuretic peptide, and troponin. There was no new organ dysfunction.

Given the exclusion of infectious processes and the daily worsening of hypoxemia, the most probable diagnosis was determined to be an AE-ILD. Therapy with methylprednisolone (1 mg/kg/day), as well as oral morphine for dyspnea, was initiated on post-admission day 4. Progressively, the patient experienced worsening gas exchange and the need for increased oxygen flow. On post-admission day 7, the use of a high-flow nasal cannula was required, at a flow rate of 60 L/min and an FiO_2_ of 80%, achieving an SpO_2_ of 85%. The patient developed mental confusion and respiratory discomfort, prompting the initiation of palliative sedation in accordance with the patient’s advance care directive. On post-admission day 15, the patient died.

The ILD category encompasses a diverse group of pulmonary disorders, characterized by varying degrees of inflammation and fibrosis, whose prognosis varies widely depending on the specific pathology.^1^ Although the diagnostic approach to ILDs and their potential comorbidities, such as lung cancer, often necessitates histopathological examination, the potential risks associated with invasive procedures like transthoracic biopsies are not negligible.^2^

Recognized as the predominant pulmonary manifestation in patients with RA, ILD affects approximately 20% of this patient population. Among these, UIP is the most commonly observed pattern.^3^ ^,^ ^4^ Notably, the risk of lung cancer is higher in individuals with RA than in those without.^5^

A rapidly progressing, life-threatening respiratory condition, AE-ILD is characterized by the emergence of new, extensive alveolar abnormalities superimposed on preexisting pulmonary fibrosis. The diagnostic criteria for AE-ILD include the presence of fibrosing ILD evident on HRCT; an acute onset or worsening of dyspnea, typically within one month; the appearance of a new bilateral ground-glass opacity or consolidation on HRCT; and clinical deterioration that cannot be fully attributed to cardiac failure or fluid overload.^6^ The 90-day mortality rate associated with AE-ILD is approximately 50% in patients with RA.^7^

A multitude of factors, including infections, air pollution, aspiration events, transfusions, medications, and pulmonary sample collection, can trigger AE-ILD.^2^ ^,^ ^8^ Procedures associated with AE-ILD include open surgical biopsy, video-assisted thoracoscopic biopsy, BAL, TTB, lung cryobiopsy, and even surgical procedures not directly involving the lungs. However, the risk factors for AE-ILD in patients with RA-ILD are not well understood.^2^

In the case presented here, we believe the most likely explanation for the respiratory deterioration and bilateral ground-glass opacities observed on the HRCT scan was that they were secondary to the TTB. That belief is based on the temporal correlation with the biopsy and the absence of infectious findings, new hemoptysis events, oncological treatments, or other invasive procedures (given that the prior bronchoscopy involved only local hemostatic measures without BAL). Although two other cases of AE-ILD secondary to TTB were reported in a retrospective study,^9^ the underlying lung disease in those cases was idiopathic pulmonary fibrosis. For the treatment of AE-ILD, the recommendation in the literature is to use a high dose of methylprednisolone (500-1000 mg per day) for three days.^8^ However, we opted for a lower dose of corticosteroid therapy because of the advanced oncologic disease.^8^ We initiated treatment only after all infectious causes had been excluded, and that delay could have had a negative impact on the outcome.

In summary, the indication for invasive procedures in patients with ILD should be approached cautiously, because various complications may arise, including acute pulmonary exacerbations.^9^ Early recognition of AE-ILD, followed by the initiation of treatment with systemic corticosteroids, is crucial given that this condition has a remarkably high 90-day mortality rate.
